# ALD-grown two-dimensional TiS_*x*_ metal contacts for MoS_2_ field-effect transistors†

**DOI:** 10.1039/d3na00387f

**Published:** 2023-08-15

**Authors:** Reyhaneh Mahlouji, Wilhelmus M. M. (Erwin) Kessels, Abhay A. Sagade, Ageeth A. Bol

**Affiliations:** a Department of Applied Physics, Eindhoven University of Technology P. O. Box 513 5600 MB Eindhoven The Netherlands r.mahlouji@tue.nl a.a.bol@tue.nl; b Department of Physics and Nanotechnology, Laboratory for Advanced Nanoelectronic Devices, SRM Institute of Science and Technology SRM Nagar, Kattankulathur 603 203 Tamil Nadu India abhaya@srmist.edu.in

## Abstract

Metal contacts to MoS_2_ field-effect transistors (FETs) play a determinant role in the device electrical characteristics and need to be chosen carefully. Because of the Schottky barrier (SB) and the Fermi level pinning (FLP) effects that occur at the contact/MoS_2_ interface, MoS_2_ FETs often suffer from high contact resistance (*R*_c_). One way to overcome this issue is to replace the conventional 3D bulk metal contacts with 2D counterparts. Herein, we investigate 2D metallic TiS_*x*_ (*x* ∼ 1.8) as top contacts for MoS_2_ FETs. We employ atomic layer deposition (ALD) for the synthesis of both the MoS_2_ channels as well as the TiS_*x*_ contacts and assess the electrical performance of the fabricated devices. Various thicknesses of TiS_*x*_ are grown on MoS_2_, and the resultant devices are electrically compared to the ones with the conventional Ti metal contacts. Our findings show that the replacement of 5 nm Ti bulk contacts with only ∼1.2 nm of 2D TiS_*x*_ is beneficial in improving the overall device metrics. With such ultrathin TiS_*x*_ contacts, the ON-state current (*I*_ON_) triples and increases to ∼35 μA μm^−1^. *R*_c_ also reduces by a factor of four and reaches ∼5 MΩ μm. Such performance enhancements were observed despite the SB formed at the TiS_*x*_/MoS_2_ interface is believed to be higher than the SB formed at the Ti/MoS_2_ interface. These device metric improvements could therefore be mainly associated with an increased level of electrostatic doping in MoS_2_, as a result of using 2D TiS_*x*_ for contacting the 2D MoS_2_. Our findings are also well supported by TCAD device simulations.

## Abstract 2

2D metallic TiS*_x_* can be grown at low temperatures using atomic layer deposition (ALD). Herein, we show that ultrathin films of 2D TiS*_x_* (∼1.2 nm) prepared by ALD can be used as contacts to 2D MoS_2_ in field-effect transistors (FETs) and improve the overall device metrics.

## Graphical abstract

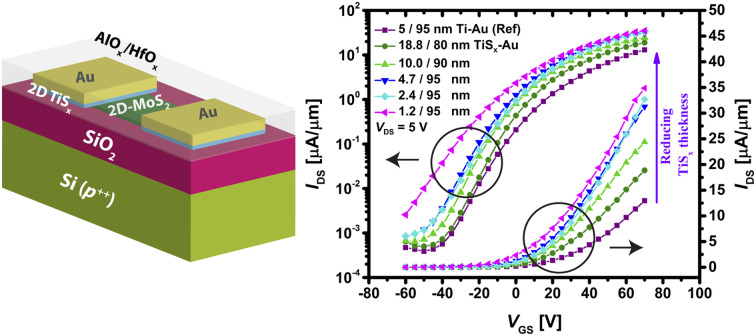

## Introduction

Transition metal di-chalcogenides (TMDCs) are a family of two-dimensional (2D)-layered materials with a chemical formula of MX_2_ (M = Mo, W, Ti, Nb, V, *etc.* and X = S, Se, Te).^1,2^ 2D TMDCs constitute a wide library of compounds ranging from semiconductors and (semi)metals to superconductors.^3^ Among semiconducting 2D TMDCs, MoS_2_ is the most widely explored material because of its abundance in nature and its outstanding electronic properties.^4,5^ Field-effect transistors (FETs) based on MoS_2_ demonstrate high current densities of 700–1135 μA μm^−1^,^6–8^ high ON/OFF current ratios in the range of 10^7^–10^9^,^5,9,10^ low subthreshold swing (SS) values close to the thermionic limit (∼60 mV dec^−1^),^11,12^ reasonably good mobility,^5,13,14^ decent reliability,^9^ relatively low variability^15^ and compatibility with conventional Si processing technologies.^16–18^ These fascinating features of MoS_2_ FETs may open new horizons for ultra-scaled nanoelectronic devices and circuits.^19,20^

Implementation of MoS_2_ or any other 2D TMDC into mainstream technology platforms is not without hurdles. 2D FETs generally suffer from high contact resistance (*R*_c_),^21^ which is still above the requirements specified by the International Roadmap for Devices and Systems (IRDS).^22^ High *R*_c_ originates from the unavoidable Schottky barrier (SB) formation and Fermi level pinning (FLP) effect at the metal–semiconductor (M–S) junctions.^23,24^ In recent years, several attempts have been made to tackle high *R*_c_ in 2D FETs, namely substitutional/chemical doping of the 2D layer,^25,26^ 2D phase engineering at the contact regions,^27^ the insertion of oxide buffer layers (Ta_2_O_3_, Al_2_O_3_) below the contacts,^28,29^ electrostatically doping the 2D channel by high-κ dielectrics,^6,10,30^ switching to edge contact device geometry (rather than using the conventional top contact device geometry)^31–34^ and utilization of semi-metal,^8^ graphene^32,35–38^ or metallic 2D TDMC contacts.^34,39,40^ To date, the lowest *R*_c_ records are in the range of 123–520 Ω μm, being held for semi-metal^8^ or graphene contacts,^37^ phase engineering the 2D layer at the contact areas,^27^ dielectric mediated charge transfer doping of the 2D channel^6^ and metallic 2D TMDC employment in edge contact device geometry.^34^

Among the above-mentioned methods for reducing *R*_c_, the usage of 2D metallic contacts, such as VS_2_,^34^ VSe_2_ ^39^ or NbS_2_,^40^ in 2D FETs has lately gained a surging interest. Conventional bulk metallic contacts are known to form covalent bonds with the 2D semiconducting layer,^41^ leading to charge redistribution at the M–S junction, work function (WF) modulations and metal-induced gap state (MIGS)^42^ formation as well as 2D electronic band-structure perturbation that altogether result in high SB/strong FLP^14,41–43^ and therefore an overall high *R*_c_. 2D metallic contacts, on the other hand, offer several advantages over the 3D bulk counterparts. First and foremost, they only weakly bind/react with 2D semiconductors. This is mainly because of the overall weak van der Waals (vdW) interactions that leads to an almost clean and flat vdW interface at the 2D–2D M–S junctions, wherein lattice matching becomes less important.^44^ Such vdW interactions are also shown to suppress MIGS and allow for an almost unperturbed 2D semiconductor electronic band-structure, weaker FLP and lower *R*_c_.^45^ Second, the WF in 2D metals can be modulated by the application of an external electric field, enabling the control of Schottky barrier height (SBH) formed at the 2D M–S junctions.^45,46^

The other challenge ahead of integrating metallic and/or semiconducting 2D TMDCs into nanoelectronic devices and circuits is their high quality and large-scale synthesis. Among the different synthesis methods, chemical vapor deposition (CVD) is shown to be one of the most promising techniques for the growth of 2D TMDCs, as it ensures the delivery of premium quality films over large areas.^13,47–51^ However, the high thermal budget that is often used in CVD may be a concern for the semiconductor industry. In addition, realization of vdW heterostructures made from 2D metals and semiconductors by using CVD, in both edge and top contact device geometries, typically involves complex procedures.^34,39,40^

In recent years, atomic layer deposition (ALD) has drawn attention for the growth of not only single layer 2D TMDCs^52–55^ but also their heterostructures (both in lateral^56^ and horizontal directions^57^). ALD is a low-temperature thin-film cyclic synthesis technique which is highly compatible with conventional Si technologies and excels in large area uniformity, thickness control down to sub-monolayer regime as well as conformality for high aspect ratio features.^58,59^

In this work, we employ ALD for the growth of both 2D metallic and 2D semiconducting layers and introduce a straightforward approach for the fabrication of 2D-based FETs. We chose TiS_*x*_ (*x* ∼ 1.8) as the contacts and MoS_2_ as the semiconducting channel material. TiS_2_ is one member of the 2D TMDC family with (semi)metallic^60^ properties. Theoretically, it has been shown that if employed as the contact electrodes, TiS_2_ forms Schottky (Ohmic) contacts with n-type (p-type) MoS_2_,^61^ due to its high WF (∼5.7 eV).^45,62,63^ In addition, it preserves the MoS_2_ intrinsic properties, meanwhile delivering high electrical conductivities.^61^ During our study, we compare ALD grown TiS_*x*_ contacts of various thicknesses with evaporated conventional Ti counterparts. We demonstrate that the fabricated MoS_2_ FETs with ∼1.2 nm thick TiS_*x*_ contacts outperform the ones with Ti contacts, as the overall MoS_2_ FET device figures of merit (*e.g.* the maximum current density (*I*_ON_), field-effect mobility (*μ*_FE_) and *R*_c_) improve when such ultrathin layers of TiS_*x*_ contacts are utilized.

## Experimental

### MoS_2_ film synthesis

A two-step approach was followed for the synthesis of MoS_2_, whereby ∼1.5 nm MoO_*x*_ was initially grown using plasma-enhanced (PE-)ALD at 50 °C,^64^ in an Oxford Instruments Plasma Technology (FlexAL) ALD reactor, on degenerately doped (*p*^++^) Si substrates that were covered with ∼87 nm SiO_2_. The as-deposited MoO_*x*_ films were then sulfurized in a home-built tube furnace, where a gas mixture of H_2_S/Ar (10%/90%) was introduced at 900 °C for 45 min, resulting in ∼1.2 nm thick MoS_2_ films. Further details of the synthesis conditions and the MoS_2_ film specifications are reported in ref. 57 and 65.

### TiS_*x*_ contact synthesis

Direct thermal ALD was employed for the growth of TiS_*x*_ contacts with various thicknesses at 100 °C. The deposition took place in the FlexAL reactor. Tetrakis (dimethyl amido) titanium (TDMAT) (Sigma-Aldrich Chemie BV, 99.999% pure) was chosen as the precursor. During the first half cycle of ALD, TDMAT was dosed into the reaction chamber for 4.2 s with Ar carrier gas, at a pressure of 80 mTorr, followed by a 20 s Ar purge step with a flow rate of 300 sccm, at the lowest achievable reaction chamber pressure (∼7 mTorr). In the second ALD half cycle, H_2_S/Ar gas mixture was introduced as the co-reactant for 30 s, with a flow rate of 10/40 sccm and at 80 mTorr, followed by another Ar purge step (with similar conditions mentioned above). More information regarding the TiS_*x*_ synthesis on SiO_2_ or on 2D TMDC substrates as well as TiS_*x*_ film specifications (*e.g.* TiS_*x*_ chemical composition, plane orientation, morphology, electrical resistivity and *etc.*) can be found in previous studies.^56,66^

### Film thickness measurements

During the PE-ALD of MoO_*x*_ and thermal ALD of TiS_*x*_, the film thicknesses were measured by *in situ* spectroscopic ellipsometry (SE) (J. A. Woollam Co., Inc. M-2000FI, 0.75–5 eV). From the obtained data, the growth per cycle (GPC) was determined. The final MoS_2_ film thickness was also verified using *ex situ* SE (J. A. Woollam Co., Inc. M-2000D, 1.25–6.5 eV). All the collected data were analyzed using complete EASE software and its embedded B-spline oscillator model.

### Device fabrication

Standard electron beam lithography (EBL) was carried out for the fabrication of back-gate MoS_2_ FETs, and PMMA was used as the electron sensitive resist. Details of the device fabrication are described in ref. 67. During the first EBL step, contact regions were defined on the PMMA coated MoS_2_. Various thicknesses of TiS_*x*_ were then grown by thermal ALD on the PMMA opening areas, in the FlexAL reactor and at 100 °C. Choosing such a low deposition temperature ensures that PMMA does not evaporate during the growth of TiS_*x*_. Immediately after the TiS_*x*_ growth, the samples were transferred into an electron beam (e-beam) evaporation chamber, where an Au layer of maximum 95 nm was deposited. The Au deposition is to facilitate probing the contacts during the electrical measurements. For the reference case, 5/95 nm of Ti/Au^67^ was e-beam evaporated in the contact openings (with similar conditions as of the Au layer on the ALD grown TiS_*x*_). Next, the lift-off process was carried out by submerging the samples in acetone overnight. For defining the channel regions and isolating the individual blocks, a second EBL step was required, followed by MoS_2_ dry etching from the opened areas using SF_6_/O_2_ plasma gas mixture in an Oxford Instruments Reactive Ion Etching (RIE) reactor. Finally, PMMA was removed in acetone, and the fabricated devices were immediately capped with 5/25 nm of thermal ALD AlO_*x*_^68^/PE-ALD HfO_*x*_,^69^ both processed at 100 °C.

### Electrical characterization

Current–voltage (*I–V*) measurements were performed in a cryogenic probe station (Janis ST-500) at a base pressure of ∼1.9 × 10^−4^ mbar and with a Keithley 4200-SCS parameter analyzer.

### Device simulations

Technology computer-aided design (TCAD) simulations were carried out using SILVACO, and standard semiconductor physics transport equation solutions were obtained by the Newton method. The simulation parameters were selected in accordance with the experimental data.

## Results and discussion

**Fig. 1:**
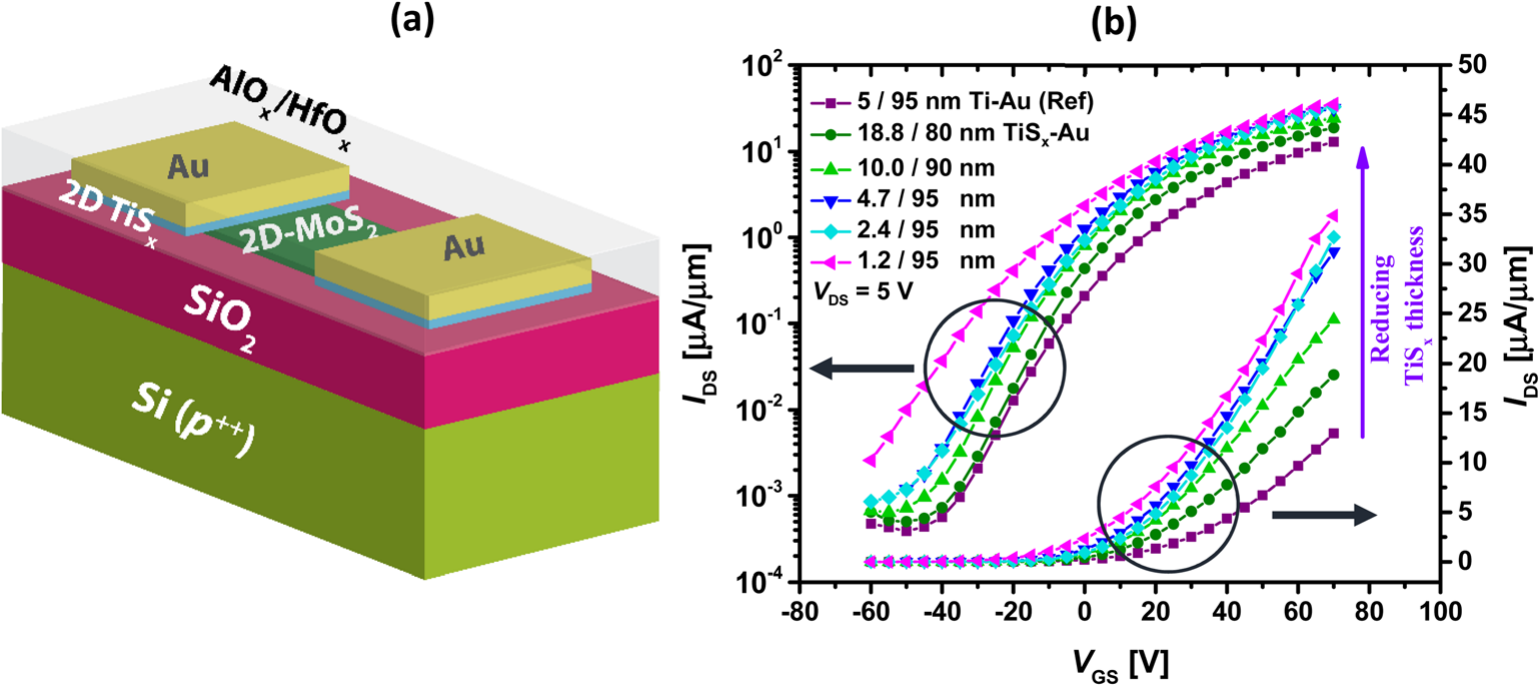
(a) Schematics of the fabricated MoS_2_ FETs, (b) measured transfer curves of the devices with series of TiS_*x*_ contact thicknesses, in both semilog and linear scales. Data for the reference sample is also provided.

Series of TiS_*x*_ thicknesses ranging from ∼20 nm down to ∼1.2 nm were grown as the contacts to MoS_2_ using thermal ALD at 100 °C. For the ease of probing the contacts during the *I–V* measurements, an Au layer of maximum 95 nm was evaporated on top of TiS_*x*_. The electrical performance of the fabricated devices were assessed and compared to a reference device, for which 5/95 nm of Ti/Au was employed as the contacts. As per a previous report,^67^ this thickness combination is found to be the most optimal for the Ti/Au stacks contacted to the ALD-based MoS_2_ films. Fig. 1(a) shows the schematics of the fabricated devices.

The transfer curves (*I*_DS_–*V*_GS_) of the MoS_2_ FETs with different TiS_*x*_ contact thicknesses are also provided in Fig. 1(b) and compared to the reference device. In all the cases, the current is measured on 500 nm long MoS_2_ channels and normalized to the device width (1 μm). At first sight, it is explicit that the entire series of TiS_*x*_-contacted devices outperform the reference case. In addition, reducing the TiS_*x*_ thickness from 20 nm down to 1.2 nm improves the overall electrical performance. The maximum ON-state current (*I*_ON_) increases to ∼35 μA μm^−1^ for the MoS_2_ FETs with the thinnest TiS_*x*_ contacts, which is nearly three times higher than that of the reference. Furthermore, the threshold voltage (*V*_T_) shifts negatively with reducing the TiS_*x*_ thickness, and the OFF-state current (*I*_OFF_) increases for the thinnest TiS_*x*_ contacts of ∼1.2 nm, both implying an increase in the MoS_2_ doping level.^12,70^ Such doping effects have also been observed in previous studies where other 2D metallic contacts (*e.g.* graphene,^36,38^ NbS_2_ ^40^ or VSe_2_ ^39^ have been utilized. We note that in general, any kind of metal (2D or 3D bulk) dopes MoS_2_ or other 2D semiconductors up to a certain extent.^41,61,70,71^

For verifying the repeatability of the observations shown in Fig. 1(b), another set of MoS_2_ FETs with series of TiS_*x*_ contact thicknesses were fabricated and characterized. Similar trends were observed for the second set upon reducing the TiS_*x*_ contact thickness. See Fig. S1(a) and (b) in the ESI† and the associated discussion.

**Fig. 2:**
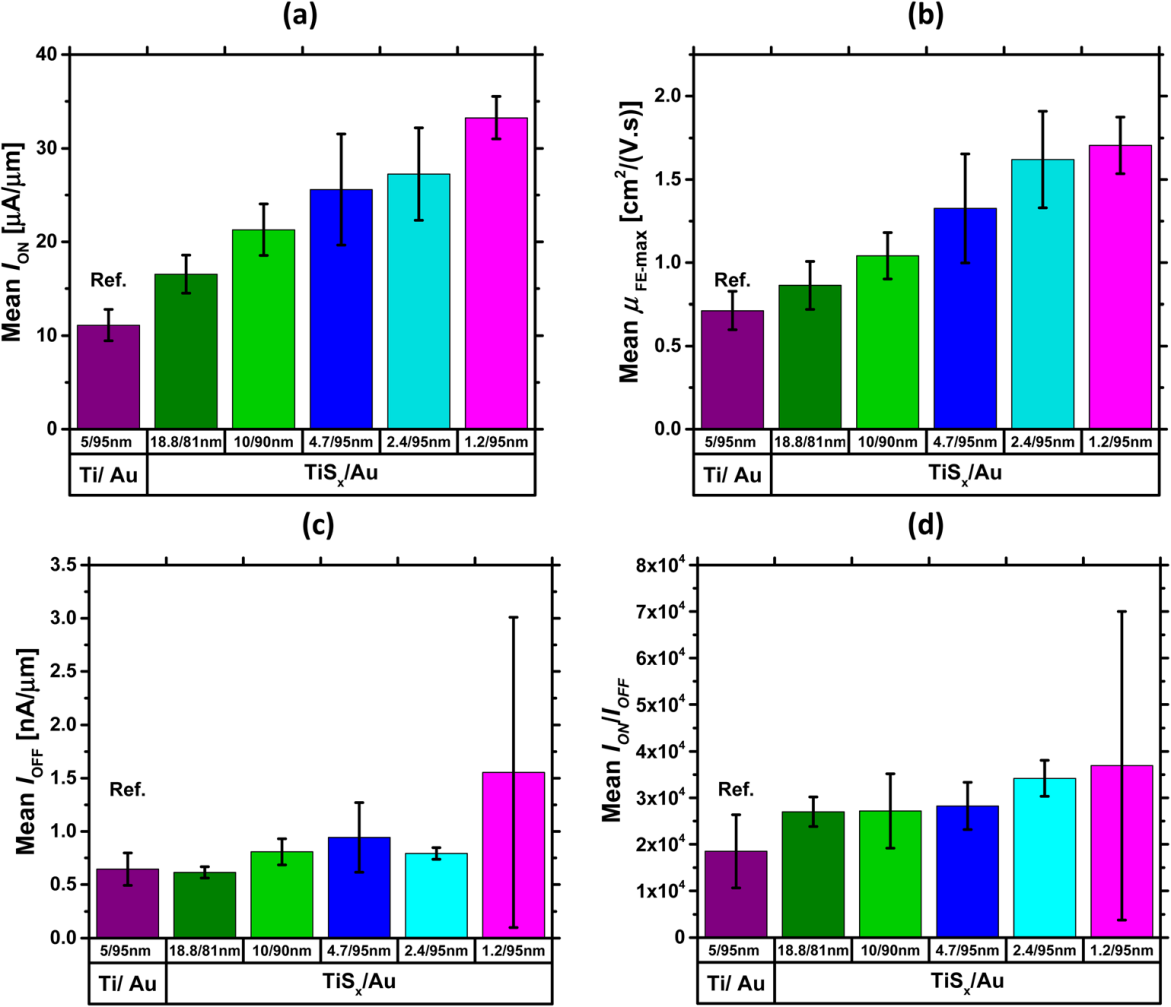
Average statistical data of (a) *I*_ON_, (b) maximum *μ*_FE_, (c) *I*_OFF_ and (d) ON/OFF current ratio for the MoS_2_ FETs with various TiS_*x*_ thicknesses, all obtained at *V*_DS_ = 5 V. Data for the reference case with Ti/Au contacts is also included.

To further gain insight into the overall electrical performance of the MoS_2_ FETs with TiS_*x*_ contacts, the average statistical data of *I*_ON_, maximum *μ*_FE_, *I*_OFF_ and ON/OFF current ratio are provided in Fig. 2(a)–(d), respectively. The presented data were obtained by measuring three-four devices on each studied sample.

In Fig. 2(a) and (b), *I*_ON_ and the maximum *μ*_FE_ show a monotonically increasing trend with reducing the TiS_*x*_ thickness. In addition, the devices with the thickest TiS_*x*_ contacts (∼20 nm) still outperform the reference devices of ∼5 nm Ti. Both values (*I*_ON_ and maximum *μ*_FE_) also increase nearly twice on average when the TiS_*x*_ thickness reduces to ∼1.2 nm. These observed device performance improvements by reducing the TiS_*x*_ contact thickness can be mainly attributed to the reduction of the interfacial tunneling barrier within TiS_*x*_, which leads to a reduction in the TiS_*x*_ overall interlayer resistance.^61^ In fact, because layers in 2D TMDCs are generally held by weak vdW forces, a gap is always present in between the individual layers. This gap acts as an interfacial tunneling barrier that scatters carriers, degrades the current and contributes to *R*_c_.^41,61,72,73^ Therefore, when the number of layers in a 2D metallic TiS_*x*_ reduces, the interfacial tunneling barrier and the resulting interlayer resistance are expected to reduce. In addition to that, the Au electrodes get closer to the MoS_2_ active layers, altogether leading to a more efficient carrier injection/collection and hence improvements in the ON-state device characteristics. The overall superior performance of the TiS_*x*_-contacted MoS_2_ FETs to the reference devices can also be associated with the reduced perturbance of the MoS_2_ electronic band-structure,^45^ when the 3D bulk metallic contacts (Ti) are replaced with the 2D TiS_*x*_ counterparts.

Fig. 2(c) shows the average trend for *I*_OFF_. As can be seen, there is no significant change in this metric with reducing the TiS_*x*_ thickness, except for when ∼1.2 nm TiS_*x*_ contacts are employed. The rise of *I*_OFF_ in this case can be associated with the increased electrostatic doping in MoS_2_, such that higher back-gate voltages are required to fully deplete the channel in the OFF-state regime. However, because *I*_OFF_ is maintained well below 2 μA μm^−1^, a similar ON/OFF current ratio in the range of 10^4^ (Fig. 2(d)) is achieved for all the studied cases. It is worthwhile mentioning that *I*_OFF_ can be further controlled if a thinner back-gate oxide (*e.g.* 30 nm SiO_2_) is employed, as thinner SiO_2_ typically leads to improved electrostatic control over the MoS_2_ channel.

Based on the analyses provided so far, the MoS_2_ FETs with ∼1.2 nm thick TiS_*x*_ contacts were found to be the most optimally operating devices. To confirm this further, the electrical performance of the second set of MoS_2_ devices with various TiS_*x*_ contact thicknesses were also statistically evaluated. See Fig. S1(a)–(d) in the ESI.† Our analyses verify that the MoS_2_ devices with ∼1.2 nm thick TiS_*x*_ contacts still lead to the most optimal performance. Therefore, they were selected for further electrical characterization.

**Fig. 3:**
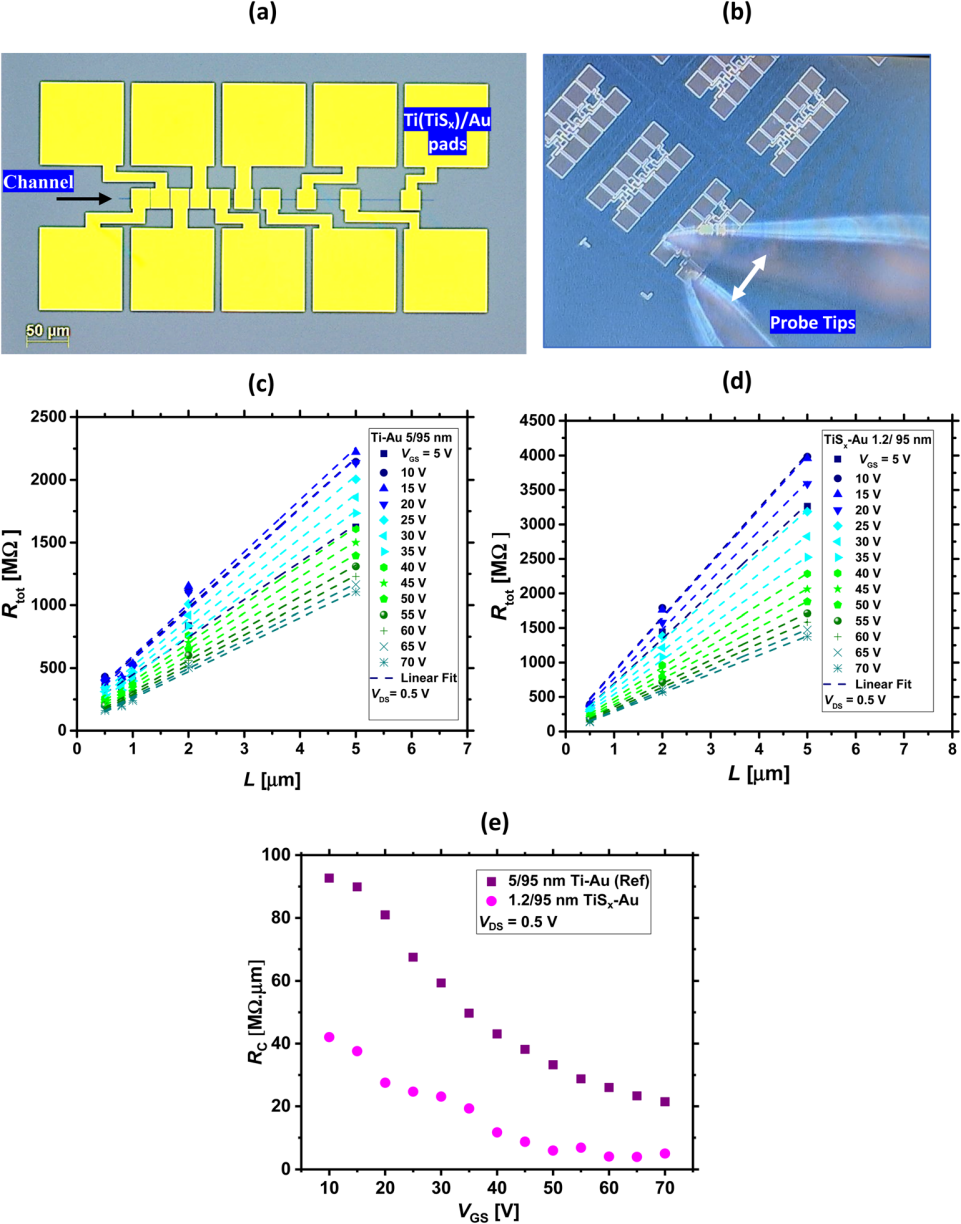
(a) Top-view TLM layout design used for *R*_c_ extractions, with a channel width of 1 μm and various channel lengths ranging from 0.5–50 μm. As indicated, yellow squares are the Ti(TiS_*x*_)/Au contact pads. (b) Optical image of the probed TLM structures used for the *I–V* measurements. The white arrow indicates the probe tips. (c) and (d) *R*_tot_*versus L* for the MoS_2_ FETs with TiS_*x*_/Au and Ti/Au contacts, respectively (at *V*_DS_ = 0.5 V and for different *V*_GS_). (e) Extracted *R*_c_ as a function of *V*_GS_ for the TiS_*x*_/Au and Ti/Au cases.

The *R*_c_ of such devices were evaluated in the next step and compared to that of the reference case. To extract *R*_c_, transfer length method (TLM) structures^74^ of various MoS_2_ channel lengths (ranging from 0.5–5 μm) were electrically measured, and the total resistance (*R*_tot_) of both TiS_*x*_ and Ti contacts were extracted from the transfer curves. Fig. 3(a) and (b) show the layout TLM design and the optical image of the probed TLM structures used for the *I–V* measurements, respectively. A low *V*_DS_ voltage (*V*_DS_ = 0.5 V) was applied for the *R*_c_ evaluations. This was to minimize the errors occurring during the *R*_c_ extraction, as the application of high *V*_DS_ (*V*_DS_ > 1 V) resulted in negative *R*_c_ and its underestimation. The *R*_c_ values were obtained using the following formula, where the dependence of the individual parameters on the applied *V*_GS_ is also included:^74,75^

1 *R*_tot_(*V*_GS_) = 2 × *R*_c_(*V*_GS_) + *R*_sh_(*V*_GS_) × (*L*/*W*)

Here, *R*_sh_ is the channel sheet resistance, and *L* and *W* are channel length and width, respectively.

Fig. 3(c) and (d) display *R*_tot_ as a function of *L* for the TiS_*x*_ contacts and the reference case, respectively, at *V*_DS_ = 0.5 V and for different *V*_GS_ values. Using these plots, *R*_c_ can be extracted.^74^ This is provided in Fig. 3(e). As can be seen, at *V*_GS_ = 70 V, the *R*_c_ for the TiS_*x*_-contacted MoS_2_ FETs is ∼5.0 MΩ μm and nearly four times smaller than that of the reference (which is 21.4 MΩ μm). These values of *R*_c_ are still higher than what is obtained for FETs fabricated from exfoliated/CVD grown highly crystalline MoS_2_, which may be due to the nanocrystalline nature of our films and their average grain size of 70 nm.^57^ However, the replacement of 3D bulk Ti contacts with the 2D TiS_*x*_ counterparts is overall beneficial in reducing *R*_c_ of the ALD-based MoS_2_ FETs.

One might also wonder about the *R*_sh_ of the ALD-based MoS_2_. It is worthwhile mentioning that for having an accurate estimation of *R*_sh_, 4-wire measurements^76^ as well as models specifically tailored for 2D polycrystalline materials^77,78^ need to be employed.

The SBH is another important factor for evaluating the contact quality in 2D-based FETs. The carrier transport across a Schottky junction can be described by thermionic emission equation modified for 2D materials:^21,79^2
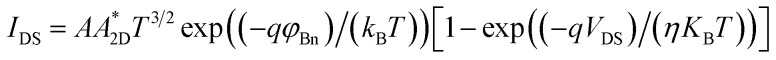
in this equation, *A* is the contact area, 
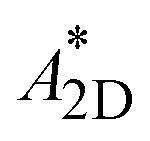
 is the 2D equivalent Richardson constant, *T* is temperature, *q* is the elementary charge magnitude, *k*_B_ is the Boltzmann constant, *φ*_Bn_ is the effective barrier height for electrons and *η* is the ideality factor. To determine *φ*_Bn_ of both TiS_*x*_ and Ti contacts to MoS_2_, low-temperature *I–V* measurements were carried out. The output data (*I*_DS_–*V*_DS_) were obtained for various *V*_GS_ (ranging from −10 V to +60 V), at seven different temperatures (180–290 K). A first order approximation of eqn (2) was used,^81^ which is expressed as the following:3
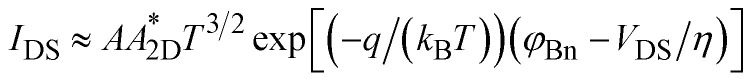


**Fig. 4:**
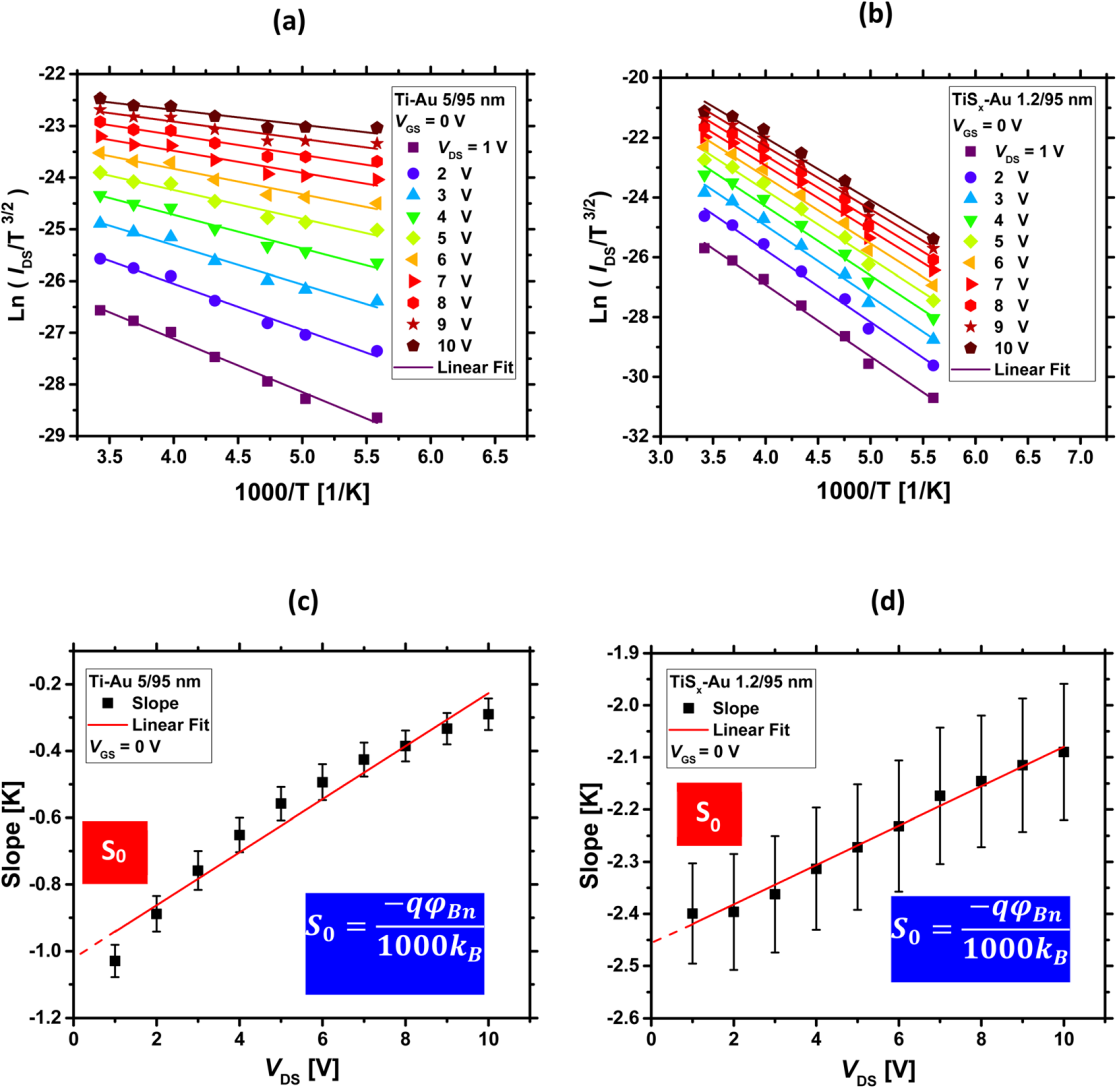
Arrhenius plots for (a) Ti/Au and (b) TiS_*x*_/Au contacts to MoS_2_ at *V*_GS_ = 0 V, (c) and (d) extracted slopes from (a) and (b) as a function of *V*_DS_, at *V*_GS_ = 0 V. The vertical axis-intercept (*S*_0_) yields *φ*_Bn_ at *V*_GS_ = 0 V.

For a fixed *V*_GS_, ln(*I*_DS_/*T*^3/2^) *versus* 1000/*T* is first plotted at each measured *V*_DS_, and a series of Arrhenius plots are obtained. This is shown in Fig. 4(a) and (b) for both Ti/Au and TiS_*x*_/Au cases, respectively. As can be seen, the acquired data are linear in each *V*_DS_. If the slope of the individual fitted lines are plotted as a function of *V*_DS_, as illustrated in Fig. 4(c) and (d) for both the TiS_*x*_ and Ti cases, the interception point with the vertical axis (*S*_0_) yields *φ*_Bn_ for a fixed *V*_GS_.^80,81^*S*_0_ is related to *φ*_Bn_ through the following equation:^81^

4 *S*_0_ = (−*qφ*_Bn_)/(1000*k*_B_)

**Fig. 5:**
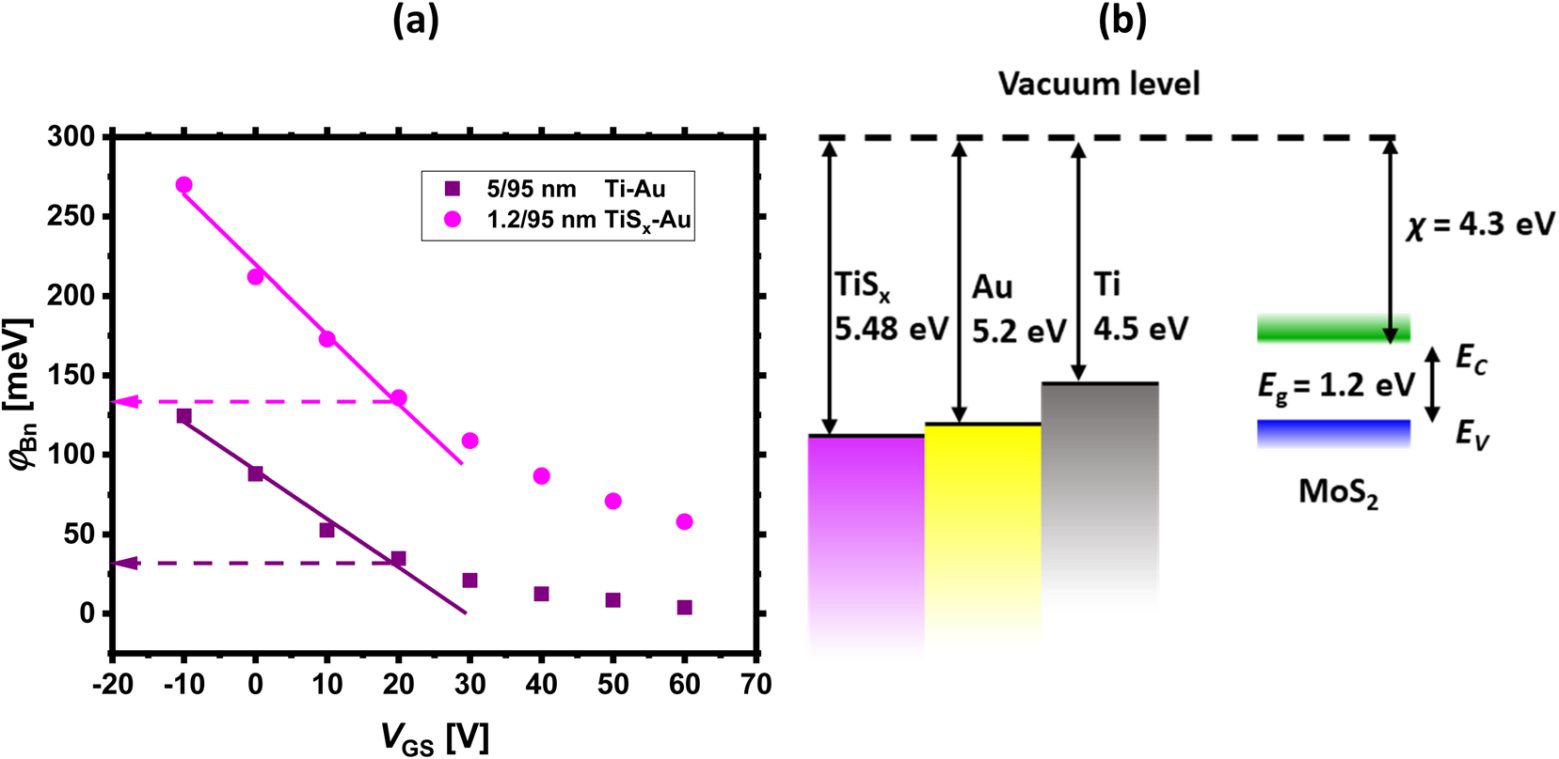
(a) Effective Schottky barrier height (*φ*_Bn_) as a function of *V*_GS_ for both Ti/Au and TiS_*x*_/Au contacts to MoS_2_ FETs. Below the flat band condition, *φ*_Bn_ reduces linearly with increasing *V*_GS_, which is marked by the fitted straight lines in both contact cases, (b) energy band diagrams of Ti, sulfur-deficient TiS_*x*_^62^ and Au with respect to MoS_2_.

If the above-mentioned extractions are repeated for each measured *V*_GS_, *φ*_Bn_ as a function of *V*_GS_ can be obtained. The final results are shown in Fig. 5(a). As evidenced from this figure, *φ*_Bn_ varies linearly at low *V*_GS_ ranges (the fitted straight line). Then, it starts to deviate from the linearity at a certain *V*_GS_. In fact, the thermionic emission equation is valid only for *V*_GS_ below the flat-band potential (*V*_FB_).^23^ Above *V*_FB_, in addition to the thermionic emission, the tunneling emission contributes to the total current, leading to the observed deviation from the linear trend.^14^

Fig. 5(a) also compares the contact metal types. For all the measured *V*_GS_, *φ*_Bn_ is higher for the TiS_*x*_ contacts (270–132 meV) than for the Ti counterparts (123–35 meV). This is expected, as TiS_*x*_ is theoretically predicted to have higher WF than Ti. To illustrate this concept, the energy band diagrams of Ti, TiS_*x*_ and Au are schematically depicted in Fig. 5(b).

Despite a higher *φ*_Bn_, the TiS_*x*_ contacts to MoS_2_ exhibit lower *R*_c_ than the Ti counterparts (Fig. 3(e)). Therefore, the observed *R*_c_ reduction in the TiS_*x*_-contacted devices is mostly associated with an increase in the MoS_2_ electrostatic doping level, which could be due to the achievement of a flat/clean interface at the junction upon replacing the 3D Ti contacts with the 2D TiS_*x*_ counterparts, such that the carriers are injected more efficiently into the MoS_2_ channel. The increase in the MoS_2_ n-type doping level is evidenced from the negative shift of *V*_T_ (Fig. 1(b)) and the slight increase of *I*_OFF_ (Fig. 1(b) and 2(c)), once the Ti contacts are replaced with ∼1.2 nm of TiS_*x*_. For high doping levels, the width of the SB reduces,^23^ and the carrier tunneling towards MoS_2_ further facilitates, leading to an overall increase in the current. In such a situation, the height of the SB (*φ*_Bn_) will have a smaller effect on the overall device performance.

Considering TiS_*x*_ contacted MoS_2_ FETs, one might find the observations contradictory to the energy band diagram analysis, as high WF metals (such as TiS_*x*_) are expected to dope MoS_2_ to p-type. Recent density functional theory (DFT) calculations by Gao *et al.*^61^ have addressed this controversy and have shown that TiS_2_ can act as both p- or n-type contact to MoS_2_, depending on the TiS_2_ number of layers and the doping concentration of both materials. TiS_2_ can also tune the barrier height at the junction, and it is predicted that for n-type 2L-TiS_2_ (∼1.2 nm) contacts to MoS_2_, the barrier height for electrons is two-times smaller than for holes. Hence, in contrast to the current band theory, it is possible to ignore p-type doping of MoS_2_ by TiS_*x*_ contacts. Similar experimental observations were also reported by Bark *et al.*^40^ when replacing 3D Mo contacts (WF ∼ 4.5 eV) with high WF 2D NbS_2_ contacts (WF ∼ 6.1 eV)^45^ in MoS_2_ FETs. These studies indicate that there is a clear distinction between 3D and 2D metals contacting 2D semiconductors.

It is also worthwhile mentioning that for ultrathin layers of 2D TiS_*x*_, quantum confinement effects start to play a role, which can affect the TiS_*x*_ electronic band structure and its alignment to that of MoS_2_ at the interface. Hence, providing a more realistic picture of the TiS_*x*_/MoS_2_ energy band diagrams may require additional DFT simulations.

**Fig. 6:**
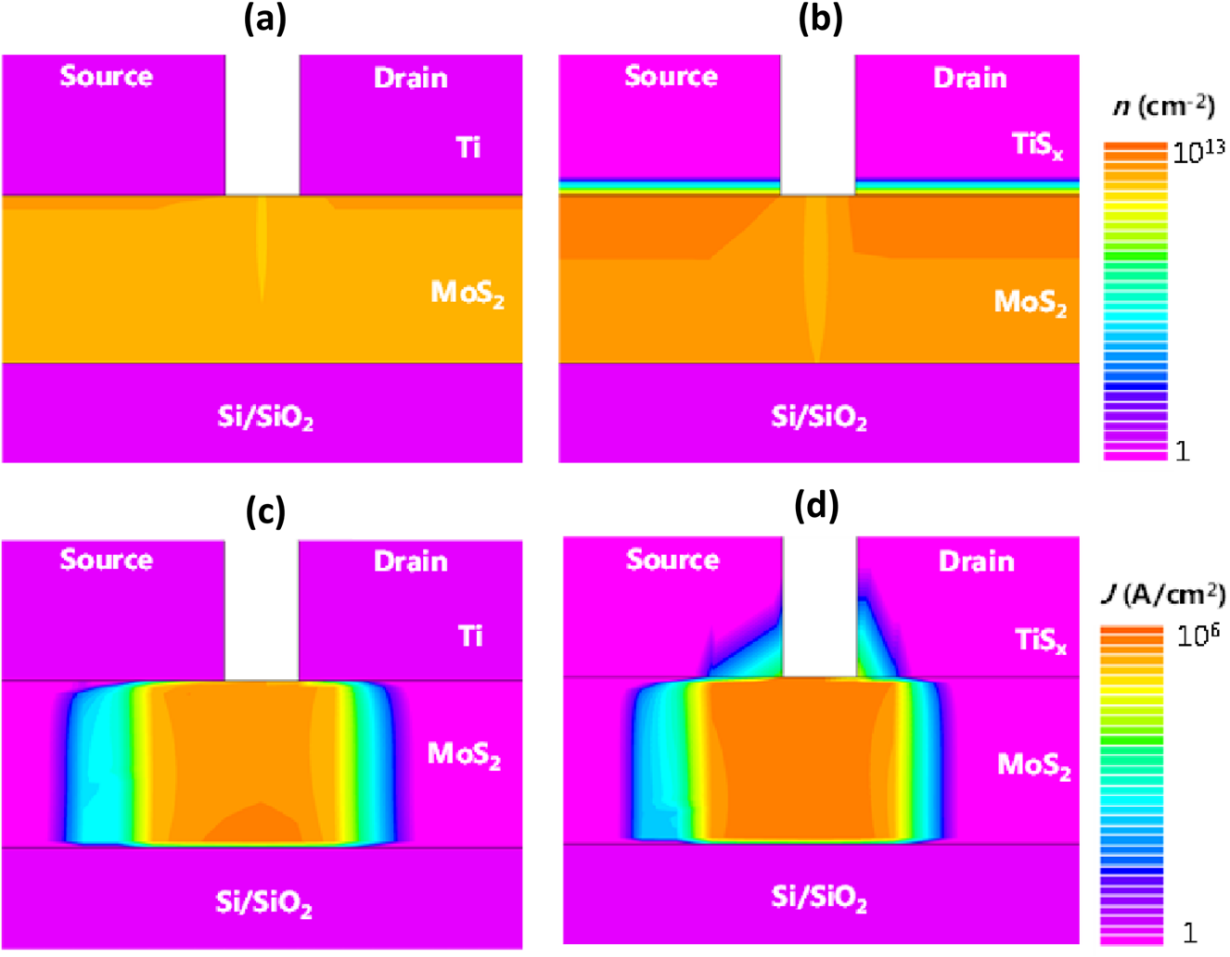
(a and b) 2D contour plots of the induced charge carrier density and (c and d) current density for Ti and TiS_2_ contacts to MoS_2_, respectively. Simulations were performed for 1.2 nm of MoS_2_, 5 nm of Ti and 1.2 nm of TiS_2_ at *V*_DS_ = 1 V and *V*_GS_ = 30 V.

To further understand the discrepancies between the 2D and 3D metals contacting a 2D TMDC semiconductor and to verify our experimental results, TCAD simulations were also performed for the 2D TiS_2_ and 3D Ti contacts to MoS_2_. The simulated device had a channel length of 500 nm and consisted of 1.2 nm of MoS_2_, 5 nm of Ti and 1.2 nm of TiS_2_. Selection of 1.2 nm TiS_2_ was because this thickness led to the most optimally performing MoS_2_ FETs in our experiments. The biasing conditions were *V*_DS_ = 1 V and *V*_GS_ = 30 V, to ensure that both device types are fully in their ON-state regime. Further details regarding the simulation parameters are provided in the ESI, Section S.3.† The resultant 2D contour plots of the gate-field-induced charge carrier density and the current density are displayed in Fig. 6(a)–(d), respectively. We note that Au contact pads are not shown in these figures.

As can be seen from Fig. 6(a) and (b), when TiS_2_ is in contact with MoS_2_, more charges are induced into the channel and MoS_2_ is doped to a higher extent, compared to the Ti case. The increase in the induced charge carrier density close to the contact regions can be distinguished by the dark orange color. As anticipated, the transverse field-induced carrier formation can be observed at the TiS_2_–MoS_2_ interface as well. Comparing Fig. 6(c) and (d), an increase in the MoS_2_ current density can be noted for the case of TiS_2_. In fact, only a small portion of TiS_2_ is actively in contact with MoS_2_, which facilitates the charge transport and subsequently leads to improved current density as well as the overall reduction of *R*_c_. The provided simulation results well confirm the experimental observations and highlight the importance of integrating 2D metallic contacts with 2D semiconductors in 2D-based FETs.

## Conclusions

To conclude, in this study, we have investigated the integration of 2D metallic TiS_*x*_ with semiconducting 2D MoS_2_ by using atomic layer deposition (ALD). We have shown that ALD grown TiS_*x*_ (*x* ∼ 1.8) contacts can improve the overall electrical performance of ALD-based MoS_2_ FETs, when employed as the contacts to polycrystalline MoS_2_ films. Based on our analyses, only ∼1.2 nm of ALD grown TiS_*x*_ is sufficient for unleashing the most from the electrical capabilities of ALD-based MoS_2_ FETs. Utilization of TiS_*x*_ contacts improved the average ON-state device characteristics and led to the achievement of *I*_ON_ as high as ∼35 μA μm^−1^. In addition and despite a higher Schottky barrier height, TiS_*x*_ contacts reduced the *R*_c_ of the fabricated MoS_2_ FETs down to ∼5.0 MΩ μm, which is nearly a quarter of what was obtained for the bulk Ti contacts (21.4 MΩ μm).

## Data availability

The data that support the findings of this study are available from the corresponding author upon request.

## Conflicts of interest

The authors declare no competing financial interest.

## Supplementary Material

NA-005-D3NA00387F-s001
