# Supplementary material for: ALD-grown two-dimensional TiSx metal contacts for MoS2 field-effect transistors
Source: Nanoscale Adv. 2023 Aug 15;5(18):4718–27. doi: 10.1039/d3na00387f (PMC10496909; doi:10.1039/d3na00387f)
Supplement: NA-005-D3NA00387F-s001 [file NA-005-D3NA00387F-s001.pdf]

Electronic Supplementary Material (ESI) for Nanoscale Advances.
This journal is © The Royal Society of Chemistry 2023

Supporting Information

ALD-Grown Two-Dimensional TiS_x Metal Contacts for MoS_2 Field-Effect Transistors

Reyhaneh Mahlouji [1], Wilhelmus M.M.(Erwin) Kessels[1], Abhay A. Sagade[2] and Ageeth A. Bol [1]

[1]Department of Applied Physics, Eindhoven University of Technology, P.O. Box 513, 5600 MB Eindhoven, The Netherlands

[2]Laboratory for Advanced Nanoelectronic Devices, Department of Physics and Nanotechnology, SRM Institute of Science and Technology, SRM Nagar, Kattankulathur 603 203, Tamil Nadu, India.

Author email: abhaya@srmist.edu.in, a.a.bol@tue.nl

S.1 *I-V* characterization of the second set of MoS_2 FETs with various TiS_x contact thicknesses

To verify the repeatability of the data shown in **Figure 1**(b) of the main text, a second set of MoS_2 FETs with various TiS_x contact thicknesses were fabricated on ~87 nm SiO_2/Si (p^{++}) substrates, and their electrical performance were compared to a reference device with 5/95 nm Ti/Au contacts. The MoS_2 channel aspect ratio was also kept similar to the initial set shown in the main text (500 nm long, 1 µm wide and ~1.2 nm thick). **Figure S1**(a) and (b) display the transfer data in linear and semilog scales, respectively. Similar to what is observed for the initial set (as shown in **Figure 1**(b) of the main text), the entire TiS_x-contacted MoS_2 FETs outperform the reference case. Furthermore, with reducing the TiS_x contact thickness, the ON-state current increases and the threshold voltage (V_T) shifts to more negative values, indicating an increase in the MoS_2 electrostatic doping.

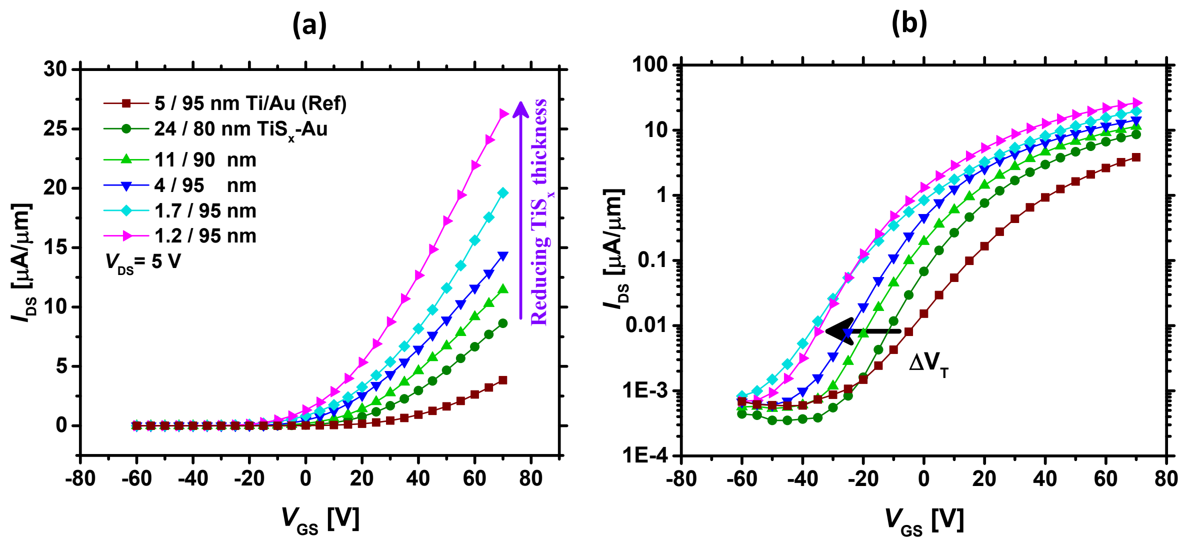

Figure S1 (a) Linear and (b) semilog transfer curves for a second set of MoS_2 FETs with various TiS_x contact thicknesses. Data for the reference case with 5/95 nm of Ti/Au contacts are also included.

S.2 Statistical analysis of the second set of MoS_2 FETs with various TiS_x contact thicknesses

Figure S2(a), (b), (c) and (d) show the average statistical data of I_{ON}, maximum μ_{FE}, I_{OFF} as well as ON/OFF current ratio, respectively. The data were obtained by measuring three-four devices on each studied sample. Comparing the results derived from the second set of devices with those from the first set (**Figure 2** in the main text) confirm that ~1.2 nm TiS_x is the most optimal thickness for the contacts to the ALD-based MoS_2 FETs.

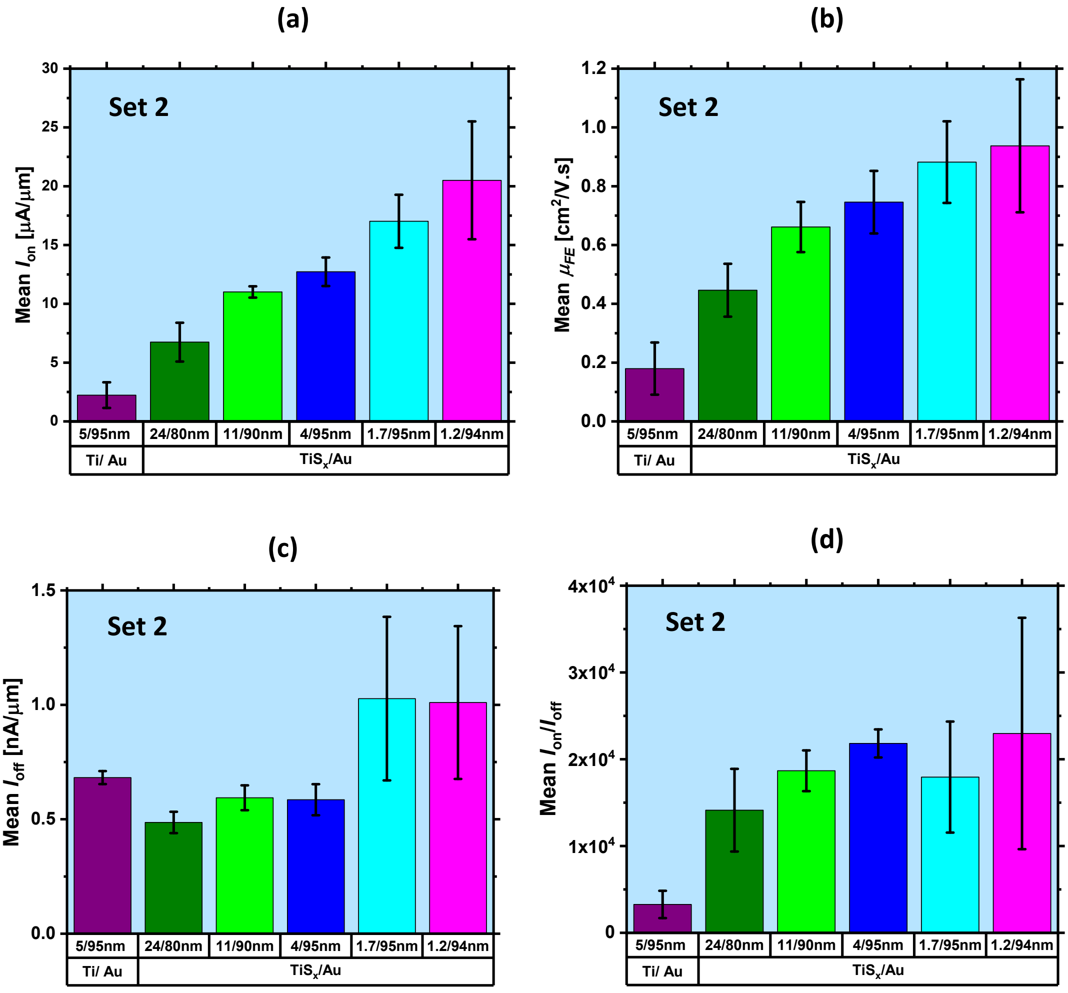

Figure S2 Average statistical data of (a) I_{ON}, (b) maximum μ_{FE}, (c) I_{OFF} and (d) ON/OFF current ratio for the second set of MoS_2 FETs with various TiS_x thicknesses, all obtained at V_{DS} = 5 V. Data for the reference case with Ti/Au contacts is also included.

S.3 TCAD simulation parameters

The TCAD simulation parameters were tuned for the device characteristics shown in **Figure 1(b)** and **Figure 2** of the main manuscript. The materials properties such as mobility, band gap (E_g), work function and etc. were chosen based on the experimental data provided in the SI (section S.1 and S.2) as well as Ref. 61 of the main manuscript.[1] Detailed thickness dependent properties reported by Gao et al.[1] in Table-1 of their manuscript were also used. **Table 1** shows some of these parameters employed in the simulations.

Table 1- Materials and properties used for TCAD simulations

**Material**	**Property**		
	Thickness [nm]	Band gap (*E*g) [eV]	Dielectric constant
MoS_2	1.2	1.4	4
TiS_2	1.2	1.23	15[2]

References

1 J. Gao and M. Gupta, *npj 2D Mater. Appl.*, 2020, **4**, 26.

2 H. El-Kouch, L. El Farh, J. Sayah and A. Challioui, *Chinese Phys. Lett.*, 2015, **32**, 096102.
